# Drug Diffusion Along an Intact Mammalian Cochlea

**DOI:** 10.3389/fncel.2019.00161

**Published:** 2019-04-26

**Authors:** Ildar I. Sadreev, George W. S. Burwood, Samuel M. Flaherty, Jongrae Kim, Ian J. Russell, Timur I. Abdullin, Andrei N. Lukashkin

**Affiliations:** ^1^Department of Medicine, Faculty of Medicine, Imperial College, London, United Kingdom; ^2^Sensory Neuroscience Research Group, School of Pharmacy and Biomolecular Sciences, University of Brighton, Brighton, United Kingdom; ^3^School of Mechanical Engineering, Institute of Design, Robotics and Optimisation, Aerospace Systems Engineering, University of Leeds, Leeds, United Kingdom; ^4^Department of Biochemistry, Biotechnology and Pharmacology, Institute of Fundamental Medicine and Biology, Kazan Federal University, Kazan, Russia; ^5^Centre for Regenerative Medicine and Devices, University of Brighton, Brighton, United Kingdom

**Keywords:** cochlea, drug delivery, salicylate, cochlear amplifier, cochlear round window

## Abstract

Intratympanic drug administration depends on the ability of drugs to pass through the round window membrane (RW) at the base of the cochlea and diffuse from this location to the apex. While the RW permeability for many different drugs can be promoted, passive diffusion along the narrowing spiral of the cochlea is limited. Earlier measurements of the distribution of marker ions, corticosteroids, and antibiotics demonstrated that the concentration of substances applied to the RW was two to three orders of magnitude higher in the base compared to the apex. The measurements, however, involved perforating the cochlear bony wall and, in some cases, sampling perilymph. These manipulations can change the flow rate of perilymph and lead to intake of perilymph through the cochlear aqueduct, thereby disguising concentration gradients of the delivered substances. In this study, the suppressive effect of salicylate on cochlear amplification via block of the outer hair cell (OHC) somatic motility was utilized to assess salicylate diffusion along an intact guinea pig cochlea *in vivo*. Salicylate solution was applied to the RW and threshold elevation of auditory nerve responses was measured at different times and frequencies after application. Resultant concentrations of salicylate along the cochlea were calculated by fitting the experimental data using a mathematical model of the diffusion and clearing of salicylate in a tube of variable diameter combined with a model describing salicylate action on cochlear amplification. Concentrations reach a steady-state at different times for different cochlear locations and it takes longer to reach the steady-state at more apical locations. Even at the steady-state, the predicted concentration at the apex is negligible. Model predictions for the geometry of the longer human cochlea show even higher differences in the steady-state concentrations of the drugs between cochlear base and apex. Our findings confirm conclusions that achieving therapeutic drug concentrations throughout the entire cochlear duct is hardly possible when the drugs are applied to the RW and are distributed via passive diffusion. Assisted methods of drug delivery are needed to reach a more uniform distribution of drugs along the cochlea.

## Introduction

The mammalian cochlea is one of the least accessible organs for drug delivery (Salt and Plontke, 2009; Rivera et al., 2012; El Kechai et al., 2015; Hao and Li, 2019). Systemic administration of many drugs, notably the most frequently used corticosteroids, and aminoglycoside antibiotics, is severely limited by the blood-labyrinth barrier (Salt and Hirose, 2018). Local intratympanic administration (Schuknecht, 1956; Bowe and Jacob, 2010) would be a preferable option for these drugs and local delivery is the only option for many old and newly emerging classes of drugs and therapies including local anesthetics, antioxidants, apoptosis inhibitors, neurotransmitters and their antagonists, monoclonal antibodies, growth factors, signaling pathway regulators, and genetic material (see Devare et al., 2018; Hao and Li, 2019 for the latest reviews). Intratympanic administration of drugs relies on their remaining in contact with the round window membrane (RW) (a membranous opening in the bony wall of the cochlea into the middle ear) long enough to allow their diffusion into the perilymph of the scala tympani (ST). The ability of drugs to pass through the RW does not, however, guarantee their sufficient distribution along the cochlear spiral. Drug distribution in the ST is limited by the low flow rate of perilymph within the cochlea and by cochlear geometry. The longitudinal flow of perilymph in the cochlea has been shown to be relatively slow, if present at all (Ohyama et al., 1988), and drug distribution in the perilymph is dominated by passive diffusion. Passive diffusion along the ST is, however, constrained because the cochlea is a relatively long and narrow tube with a cochlear cross-section that decreases gradually from the RW at the base to the apex. It is in the cochlear apex where human speech processing is initiated (e.g., Nuttall et al., 2018) and where drug delivery to the cochlea has greatest potential therapeutic and socioeconomic impact.

However, direct measurements of the distribution of marker ions and contrasting agents (Salt and Ma, 2001; Haghpanahi et al., 2013), corticosteroids (Plontke et al., 2008; Creber et al., 2018) and antibiotics (Mynatt et al., 2006; Plontke et al., 2007a) or measurements of the physiological effects of drugs (Chen et al., 2005; Borkholder et al., 2010) demonstrated that the concentration of substances applied to the RW was much higher in the cochlear base than in the apex. These measurements, however, involved perforating the cochlear bony wall and, in some cases, sampling perilymph. These manipulations can change the flow rate of perilymph (Ohyama et al., 1988; Salt and Ma, 2001) and lead to the intake of cerebrospinal fluid through the cochlear aqueduct (Salt et al., 2003), thereby disguising concentration gradients of the delivered substances.

A few studies investigated the distribution of substances applied to the RW in the intact cochlea without breaking cochlear boundaries. This was done mainly in morphological studies investigating the distribution of dexamethasone and other substances along the cochlea after their intratympanic administration (Saijo and Kimura, 1984; Imamura and Adams, 2003; Hargunani et al., 2006; Grewal et al., 2013). While these studies confirmed the existence of base-to-apex gradients, the actual concentrations of substances along the cochlea were not measured. Borkholder et al. (2014) measured the threshold elevation of distortion product otoacoustic emissions (DPOAE) produced by primary tones of different frequencies after intratympanic application of salicylate. Salicylate affects cochlear amplification in a concentration-dependent manner but the DPOAE is a non-linear phenomenon and the dependence of DPOAE thresholds on the primary tone level and cochlear amplification is complex (Lukashkin et al., 2002). As a result, salicylate concentrations along the cochlear spiral cannot be easily derived from the DPOAE threshold elevations.

The purpose of the current study is to quantify drug diffusion from the RW along an intact guinea pig cochlea, to identify the factors that limit passive drug diffusion along the cochlea, and to analyse possible solutions to overcome these limitations. Salicylate was used as a model drug with well-characterized physiological effects. A mathematical model, which includes a diffusion component and a biophysical component describing the action of salicylate on the cochlear amplifier was validated using experimental data and used to assess the distribution of substances along the human cochlea.

## Materials and Methods

### Animals

Pigmented guinea pigs of similar weight (350–360 g) were anesthetized with the neurolept anesthetic technique (0.06 mg/kg body weight atropine sulfate s.c., 30 mg/kg pentobarbitone i.p., 500 μl/kg Hypnorm i.m.). Additional injections of Hypnorm were given every 40 min. Additional doses of pentobarbitone were administered as needed to maintain a non-reflexive state. The heart rate was monitored with a pair of skin electrodes placed on both sides of the thorax. The animals were tracheotomized and artificially respired, and their core temperature was maintained at 38°C with a heating blanket and a heated head holder. All procedures involving animals were performed in accordance with UK Home Office regulations with approval from the local ethics committee.

### Signal Generation and Recording

The middle ear cavity of the ear used for the measurements was opened to reveal the RW. Compound action potentials (CAPs) of the auditory nerve were measured from the cochlear bony ridge in the proximity of the RW membrane using Teflon-coated silver wire coupled to laboratory designed and built extracellular amplifier (James Hartley). Thresholds of the N1 peak of the CAP were estimated visually using 10 ms tone stimuli at a repetition rate of 10 Hz.

For acoustic stimulation, sound was delivered to the tympanic membrane by a closed acoustic system comprising two Bruel and Kjaer 4134 ½″ microphones for delivering tones and a single Bruel and Kjaer 4133 ½″ microphone for monitoring sound pressure at the tympanum. The microphones were coupled to the ear canal via 1 cm long, 4 mm diameter tubes to a conical speculum, the 1 mm diameter opening of which was placed about 1 mm from the tympanum. The closed sound system was calibrated *in situ* for frequencies between 1 and 50 kHz. Known sound pressure levels were expressed in dB SPL re 2 × 10^−5^ Pa.

All acoustic stimuli in this work were shaped with raised cosines of 0.5 ms duration at the beginning and at the end of stimulation. White noise for acoustical calibration and tone sequences for auditory stimulation were synthesized by a Data Translation 3010 board at 250 kHz and delivered to the microphones through low-pass filters (100 kHz cut-off frequency). Signals from the acoustic measuring amplifier (James Hartley) were digitized at 250 kHz using the same board and averaged in the time domain. Experimental control, data acquisition, and data analysis were performed using a PC with programmes written in MATLAB (The MathWorks. Inc. 2018a).

Five microliters of sodium salicylate solution (either 100 mM in experiments on salicylate diffusion in the ST or 1M in experiments with complete block of the cochlear amplifier) in Hanks' Balanced Salt Solution were placed on the RW using pipettes. The solution was removed from the RW using paper wicks to observe the wash out effect.

### Model Overview

#### Diffusion and Clearing Equation

**Figure 1:**
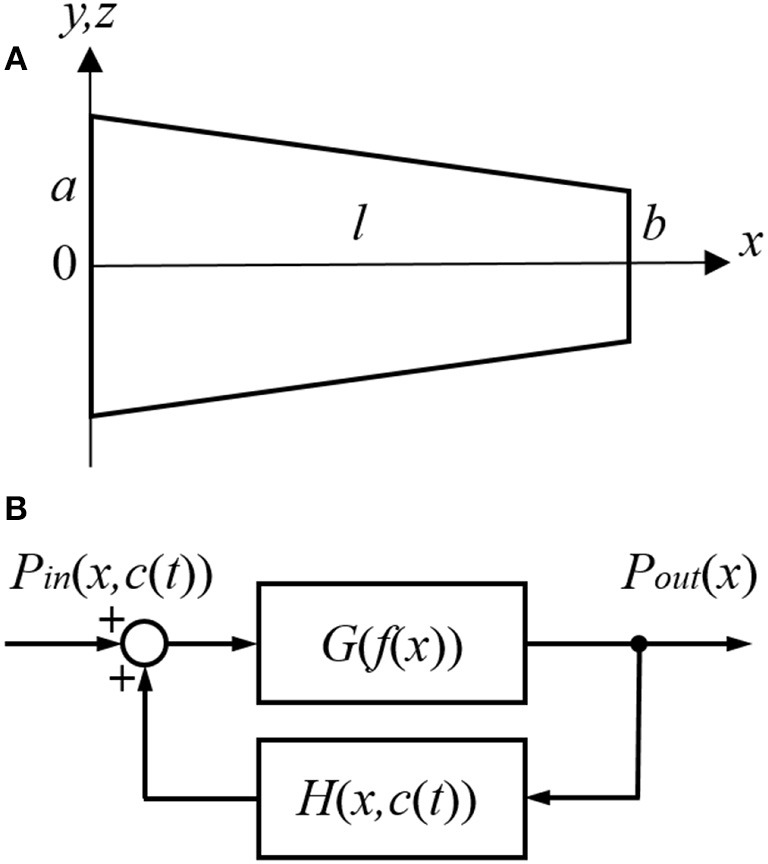
Schematic presentation of **(A)** the scala tympani (ST) approximated by a tube of decreasing diameter and **(B)** the cochlear amplifier modeled as a system with positive feedback provided by the outer hair cells (OHCs).

For the purpose of modeling, the ST is approximated by a tube with a decreasing diameter similar to that described in previous models, for example by Plontke et al. (2007b) (Figure 1A). The radii of the tube, *r*(0) and *r*(*l*), are equal to *a* and *b* at *x* = 0 and *x* = *l*, respectively, where *l* is the ST length. All the dimensions are known (Thorne et al., 1999) and symmetry along *y* and *z* axes is assumed. Zero longitudinal perilymph flow in the compartment is assumed (Ohyama et al., 1988) and only the passive diffusion of a drug (salicylate) with diffusion coefficient *k*_*d*_ is considered. In addition to diffusion, there is also clearing of the drug characterized by the clearing coefficient *k*_*c*_. This clearing can be represented simply as a leak through the scala boundary (e.g., loss to the vasculature and tissues, and to other cochlear compartments). The diffusion and clearing processes are assumed to be completely independent. Because the tube radius is much smaller than its length, i.e., *r*(*x*) ≪ *l* for all *x* in [0, *l*], only diffusion along *x* axis is considered and the concentration *c*(*x, t*) within each cross-section for a fixed instance *t* is assumed to be constant, i.e., it does not change along the *y* axis. If the area of the cross-section is *S*(*x*) then the diffusion can be described by the following partial differential equation (see Appendix for detailed derivation):

(1) $$\begin{array}{l} \frac{dc\left(x,t\right)}{dt}=\frac{1}{S\left(x\right)}\cdot \frac{d}{dx}\left(S\left(x\right)\cdot k_{d}\cdot \frac{dc\left(x,t\right)}{dx}\right)-c\left(x,t\right)\cdot \frac{2k_{c}}{r\left(x\right)}, \end{array}$$

with the boundary conditions

(2) $$\begin{array}{lll} c\left(0,t\right) & = & c_{rw}, \end{array}$$

(3) $$\begin{array}{lll} k_{d}\frac{dc\left(l,t\right)}{dx} & = & 0 \end{array}$$

and initial conditions

(4) $$\begin{array}{lll} c\left(0,0\right) & = & c_{rw};x=0, \end{array}$$

(5) $$\begin{array}{lll} c\left(x,0\right) & = & 0;x>0. \end{array}$$

The diffusion coefficient *k*_*d*_ is known (Lide, 2002) but the clearing coefficient *k*_*c*_ is unknown. The ratio of the diffusion and clearing coefficients can, however, be found via fitting the experimental data. The physical meaning of *k*_*d*_/*k*_*c*_ can be described as the ratio between the amount of substance that diffuses through a unit surface normal to the direction of diffusion for a unit concentration gradient and the amount of drug that is cleared through a unit surface normal to the direction of substance exit for a unit substance concentration, both for unit time duration. The diffusion/clearing equation was validated using experimental data on the physiological effect of salicylate on the CAP thresholds. Because the salicylate concentrations could not be directly inferred from the physiological effect of salicylate, a biophysical element of the model was developed allowing calculations of the salicylate concentrations along the cochlea.

#### Link Between Position and Frequency

The dependence between frequency of stimulation *f* and frequency position along the length *x* of the basilar membrane for the guinea pig cochlea is defined by the Greenwood equation (Greenwood, 1990)

(6) $$\begin{array}{l} f\left(\tilde{x}\right)=A\cdot \left(10^{\alpha \tilde{x}}-\beta \right), \end{array}$$

where *A* = 0.35, α = 2.1/18.5, β = 0.85 and $\tilde{x}=l-x$ meaning that the starting point for $\tilde{x}$ in Greenwood (1990) is at the apex and not the base of the cochlea, as in this study.

#### Cochlear Amplifier

The cochlear amplifier is represented by a positive feedback system (Figure 1B) with feedback gain *H*(*x, c*(*t*)) due to force generation by the OHCs (Mountain et al., 1983; Yates, 1990; Lukashkin and Russell, 1999). The following assumptions are made for a small signal, linear regime:

1. The CAP threshold is observed for different sound pressure *P*_*in*_(*x, c*(*t*)) at the tympanum but for the same BM displacements, i.e., for the same constant pressure *P*_*out*_(*x*) at the BM for any given frequency/place *x* during manipulations with the cochlear amplifier. The assumption is based on good correspondence between neural and BM thresholds at the CF (Ruggero et al., 2000; Temchin et al., 2008).
2. Feedback gain *H*(*x, c*(*t*)) is proportional to the outer hair cell (OHC) force *F*(*x, c*(*t*)) for any given frequency/place in the cochlea
  (7) $$\begin{array}{l} H\left(x,c\left(t\right)\right)=\alpha \cdot F\left(x,c\left(t\right)\right), \end{array}$$
  where α is the gain constant. The initial feedback gain *H*(*x*, 0) for any frequency/place before application of salicylate can be found empirically (see below).
3. Salicylate changes only feedback gain *H*(*x, c*(*t*)) through changes in *F*(*x, c*(*t*)).
4. In line with other modeling studies (e.g., Meaud and Grosh, 2014; Ni et al., 2016), it is assumed that pressure/displacement at the BM is a linear combination of the passive BM response due to acoustic stimulation and active response due to the OHC forces.

The link between local salicylate concentration *c*(*x, t*) and reduction in force *FR*(*x, c*(*t*)) generated by the OHCs can be described by the Hill function (Hallworth, 1997)

(8) $$\begin{array}{l} FR\left(x,c\left(t\right)\right)=V_{\max}\cdot \frac{c{\left(x,t\right)}^{n}}{k^{n}+c{\left(x,t\right)}^{n}}, \end{array}$$

where *V*_max_ = 0.71629, *k* = 0.101, and *n* = 0.983.

The reduction in force is linked to the force before *F*(*x*, 0) and after *F*(*x, c*(*t*)) salicylate application as

(9) $$\begin{array}{l} FR\left(x,c\left(t\right)\right)=\frac{F\left(x,0\right)-F\left(x,c\left(t\right)\right)}{F\left(x,0\right)}=1-\frac{F\left(x,c\left(t\right)\right)}{F\left(x,0\right)} \end{array}$$

or,

(10) $$\begin{array}{l} F\left(x,c\left(t\right)\right)=F\left(x,0\right)\cdot \left(1-FR\left(x,c\left(t\right)\right)\right). \end{array}$$

It can be written for any given frequency/place before salicylate application at *t* = 0 (Figure 1B)

(11) $$\begin{array}{l} \frac{P_{out}\left(x\right)}{P_{in}\left(x,0\right)}=\frac{G}{1-G\cdot H\left(x,0\right)}, \end{array}$$

where *G* is the open loop gain. Similarly, at time *t* after salicylate application

(12) $$\begin{array}{l} \frac{P_{out}\left(x\right)}{P_{in}\left(x,c\left(t\right)\right)}=\frac{G}{1-G\cdot H\left(x,c\left(t\right)\right)}. \end{array}$$

Dividing (11) by (12) and taking into account (7), it could be written

(13) $$\begin{array}{l} \frac{P_{in}\left(x,c\left(t\right)\right)}{P_{in}\left(x,0\right)}=\frac{1-G\cdot \alpha \cdot F\left(x,c\left(t\right)\right)}{1-G\cdot \alpha \cdot F\left(x,0\right)}. \end{array}$$

Substituting *F*(*x, c*(*t*)) from (10) into (13) and using (7), one can obtain

(14) $$\begin{array}{l} \frac{P_{in}\left(x,c\left(t\right)\right)}{P_{in}\left(x,0\right)}=\frac{1-G\cdot H\left(x,0\right)\cdot \left(1-FR\left(x,c\left(t\right)\right)\right)}{1-G\cdot H\left(x,0\right)}. \end{array}$$

The left part of (14) is measured in the experiment. *FR*(*x, c*(*t*)) is calculated using the Hill function (8) with *c*(*x, t*) in this equation being calculated using the diffusion/clearing equation (1).

An analytical form of empirical dependence *H*(*x*, 0), i.e., feedback gain before salicylate application for different frequencies/locations, can be obtained as follows. Feedback from the OHCs can be completely blocked in experiments using a high concentration of salicylate. In this case *H*(*x, c*(*t*)) = 0 in (12) and the transfer function of the feedback system (Figure 1B) is equal to the open loop gain *G*. Then similar to (11) and (12)

(15) $$\begin{array}{l} \frac{P_{out}\left(x\right)}{P_{inBlock}\left(x\right)}=G, \end{array}$$

where *P*_*inBlock*_(*x*) is the sound pressure required to produce a response from the auditory nerve in preparations where the cochlear amplifier is completely blocked, and it does not depend on time. Dividing (11) by (15) and rearranging gives the following equation

(16) $$\begin{array}{l} H\left(x,0\right)=\frac{1}{G}\cdot \left(1-\frac{P_{in}\left(x,0\right)}{P_{inBlock}\left(x\right)}\right), \end{array}$$

where *P*_*inBlock*_(*x*)/*P*_*in*_(*x*, 0) is measured in separate experiments.

*G* has frequently been assumed to be constant along the cochlea (e.g., Mountain et al., 1983; Yates, 1990; Lukashkin and Russell, 1999). In spite of the special design of the cochlea, which minimized energy losses when the BM traveling wave moves from the base to apex (Jones et al., 2013), some energy dissipation is still expected during wave propagation in a viscous environment. To account for energy losses, we assumed a simple linear dependence of the open loop gain *G*(*f*(*x*)) on frequency

(17) $$\begin{array}{l} G\left(f\left(x\right)\right)=s\cdot f\left(x\right)+i, \end{array}$$

where *s* is the slope and *i* is the intercept defined as *i* = 1 − *s* · *f*_*l*_, with *f*_*l*_ = 49.9165 kHz specifying the upper frequency limit of linear dependence for *G*(*f*(*x*)). Hence, *G*(*f*(*x*)) effectively depends only on a single parameter *s*, which could be found by fitting the experimental data.

#### Initial Model Parameters

Ratio *P*_*inBlock*_(*x*)/*P*_*in*_(*x*, 0) was measured as a function of frequency *f*. Equation (6) shows how this frequency can be converted to a coordinate. An arbitrary Hill type function

(18) $$\begin{array}{l} \frac{P_{inBlock}}{P_{in}}\left(f\right)=m_{1}\frac{f^{m_{2}}}{m_{3}^{m_{2}}+f^{m_{2}}}+m_{4} \end{array}$$

was fitted to the experimental data with 20log_10_ transformation for dB using the Genetic Algorithm (GA) tool in MATLAB (The MathWorks. Inc. 2018a) (initial local fit). The obtained *m*_1_, *m*_2_, *m*_3_, and *m*_4_ (Table 1) were then used for the later optimization procedures described below (final global fit). The feedback gain before application of salicylate *H*(*x*, 0) = *H*(*f*(*x*), 0) was obtained according to (16) and (18) as

(19) $$\begin{array}{l} H\left(f,0\right)=\frac{1}{G}\cdot \left(1-1/\left(m_{1}\frac{f^{m_{2}}}{m_{3}^{m_{2}}+f^{m_{2}}}+m_{4}\right)\right). \end{array}$$

Initial values for the all parameters used in the model before the optimization procedure are shown in Table 1.

**Table 1:** Model parameter values.

**Parameter**	**Unit**	**Initial value**	**Optimized value**	**Source of the initial value**
*a*	mm	0.56	Fixed	Thorne et al., 1999
*b*	mm	0.18	Fixed	Thorne et al., 1999
*l*	mm	18–19	19	Thorne et al., 1999
*k*_*d*_	mm^2^/s	0.959e-3	Fixed	Lide, 2002
*ratio* = *k*_*d*_/*k*_*c*_	mm	1–10	1.6968	Initial guess
*c*_*rw*_	mM	100	Fixed	Experiment
*A*	kHz	0.35	Fixed	Greenwood, 1990
α	1/mm	2.1/18.5	Fixed	Greenwood, 1990
β	–	0.85	Fixed	Greenwood, 1990
*k*	mM	0.101	Fixed	Hallworth, 1997
*n*	–	0.983	Fixed	Hallworth, 1997
*V*_max_	–	0.71629	Fixed	Hallworth, 1997
*m*_1_	–	1011.2	Fixed	Experiment
*m*_2_	–	8.1406	Fixed	Experiment
*m*_3_	kHz	3.4816	Fixed	Experiment
*m*_4_	–	31.686	Fixed	Experiment
*s*	1/kHz	0–0.0261	0.00014742	Initial guess

#### Optimized Model Parameters

Equation (14) with 20log_10_ transformation for dB was solved in MATLAB (The MathWorks. Inc. 2018a) using pdepe solver for partial differential equations and fitted to the entire set of experimental data for all frequencies and salicylate concentrations using Genetic Algorithm (GA) tool in MATLAB (The MathWorks. Inc. 2018a). The sum of squared errors

$$\begin{array}{l} SE=\overset{n}{\underset{i=1}{\sum }}{\left(M_{i}-E_{i}\right)}^{2} \end{array}$$

was used as a cost function for minimization, where *M* are the model predictions and *E* are the experimental data for points *i* = 1…*n*. It is worth noting that only three model parameters were fitted during the global fit/optimization. These parameters are the cochlear length *l*, *k*_*d*_/*k*_*c*_ ratio and slope *s* of the open loop gain *G*(*f*(*x*)).

## Results

### Cochlear Amplifier Gain

**Figure 2:**
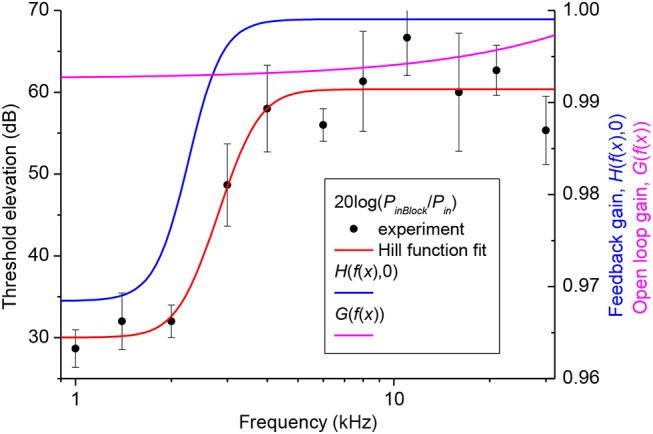
Elevation of CAP thresholds after complete block of the cochlear amplifier (left Y-axis) and corresponding value of the open loop and feedback gain (right Y-axis). Black circles show the experimental values of threshold elevation (mean ± SD, *n* = 3). Red curve indicates fit of the experimental data points by Equation (18). Related values of the parameters *m*_1_, *m*_2_, *m*_3_ and *m*_4_ are given in Table 1. Value of the open loop (magenta curve) and feedback (blue curve) gains after the final global optimization procedure were calculated using Equations (17) and (19), respectively, with the optimized value of parameter *s* (Table 1).

Gain of the cochlear amplifier and corresponding feedback gain of the model, *H*(*x*, 0) = *H*(*f*(*x*), 0) [equation (16)] was determined empirically from elevation of the CAP thresholds after application of 1M salicylate solution to the RW which caused a consistent and steady increase in threshold over the entire frequency range (Figure 2, black circles). Values for *m*_1_, *m*_2_, *m*_3_, and *m*_4_ (Table 1) were determined through fit of the experimental data points by equation (18) (Figure 2, red curve) using the Genetic Algorithm (GA) tool in MATLAB (The MathWorks. Inc. 2018a). These values were used for the general optimization procedure performed at later stages.

### Distribution of Salicylate Along the Guinea Pig Cochlea

**Figure 3:**
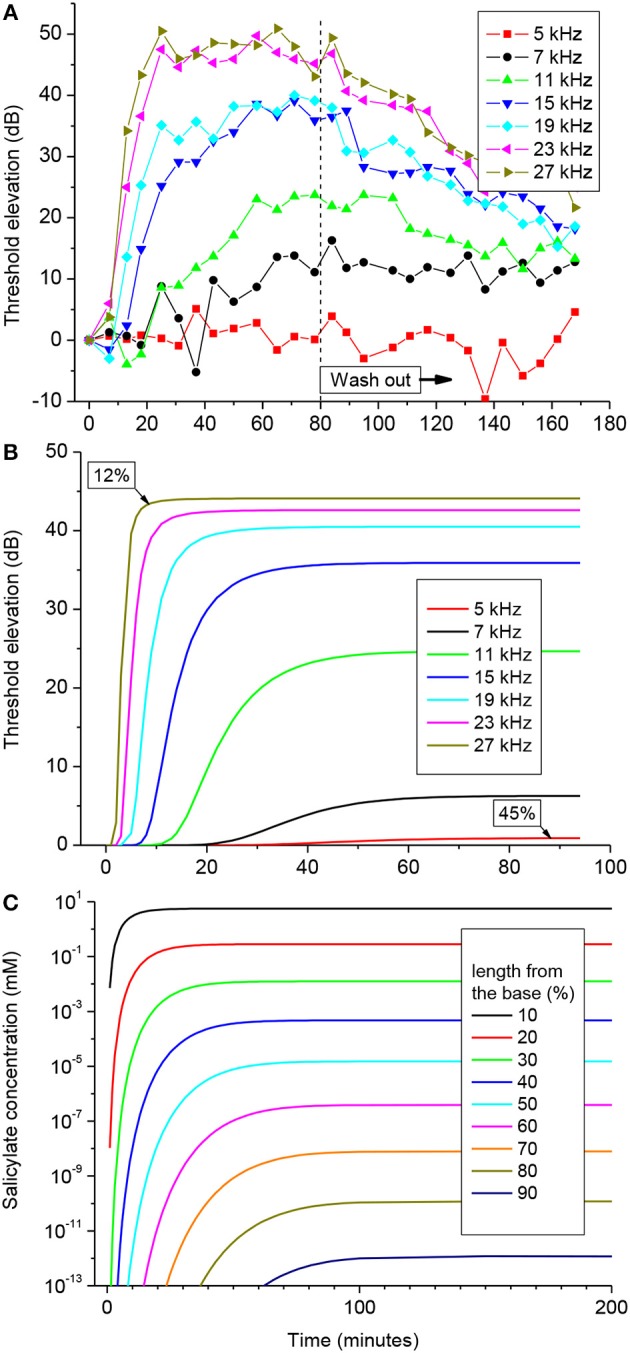
CAP threshold elevation **(A,B)** and salicylate distribution **(C)** in the guinea pig cochlea after application of 100 mM of salicylate solution to the RW at time = 0. **(A)** Representative example of the CAP threshold elevation in a single preparation. Salicylate was washed out after 80 min of application. **(B)** Combined best fit of the entire set of experimental data on CAP threshold elevation for five preparations (Figure S1) using the parameter optimization procedure. Labels indicate percentage of the total cochlear length from the base. **(C)** Salicylate concentration along the cochlear length calculated using the optimized values of the model parameters (Table 1).

One hundred mM solution of salicylate applied to the RW caused a rapid increase followed by saturation of CAP thresholds for high frequency tones (Figure 3A). CAP threshold increase for tones of lower frequencies was observed after an initial delay and did not reach saturation during the time of observation. Any changes in CAP threshold due to application of salicylate were below the noise floor of measurements for tone frequencies lower than 5 kHz, which corresponds to approximately the apical 55% of cochlear length [(Greenwood, 1990); equation (6)]. A partial recovery of the CAP threshold was observed after salicylate solution was washed out from the RW confirming that threshold elevation during application of salicylate was due to specific action of salicylate and not because of general deterioration of preparations.

Pooled data from five animals were used to find an optimized set of the model parameters via fitting the entire set of experimental data using the Genetic Algorithm (GA) tool in MATLAB (The MathWorks. Inc. 2018a) (see Materials and Methods). The combined best fit to the entire set of experimental data for the optimized set of parameters is illustrated in Figure 3B. Figure S1 shows the same plots for each of the frequencies along the corresponding experimental data. It is worth noting, that the optimization procedure was performed over the entire experimental set in order to fit the data for all the experimental frequencies simultaneously (Figure S1). The optimized set of model parameters (Table 1) found due to the general optimization procedure was used to predict cochlear responses and concentrations of salicylate (Figure 3C) along the entire cochlear length and over arbitrary time duration.

The absence of CAP threshold changes at frequencies below 5 kHz was due to poor diffusion of salicylate from the RW into the cochlear apex. It required increasingly longer times for the salicylate concentration to reach steady-state in the more apical regions of the cochlea, but at 90% of cochlear length (10% from the apex), salicylate concentration was about 12 orders of magnitude smaller than at the base even at steady-state (Figure 3C). The model suggests that this steep concentration gradient is due mainly to the fast clearing of salicylate from the ST which is reflected in the small *k*_*d*_/*k*_*c*_ ratio found in the optimization (Table 1). Because the flux *J* is proportional to the concentration gradient [equation (A1)], changes in salicylate concentration at the RW will not lead to changes in the concentration gradient between the cochlear base and apex. In this case all steady-state curves for different concentrations of salicylate at the RW are scaled versions of each other (data not shown). Hence, for a specific substance (i.e., for specific diffusion (*k*_*d*_) and clearing (*k*_*c*_) coefficients) and for a given cochlear geometry, the ratio of steady-state concentrations at the base, and apex of the cochlea is a constant and does not depend on substance concentration at the RW. This was further assessed for the human cochlea.

### Diffusion of an Arbitrary Substance in the Human Cochlea

**Figure 4:**
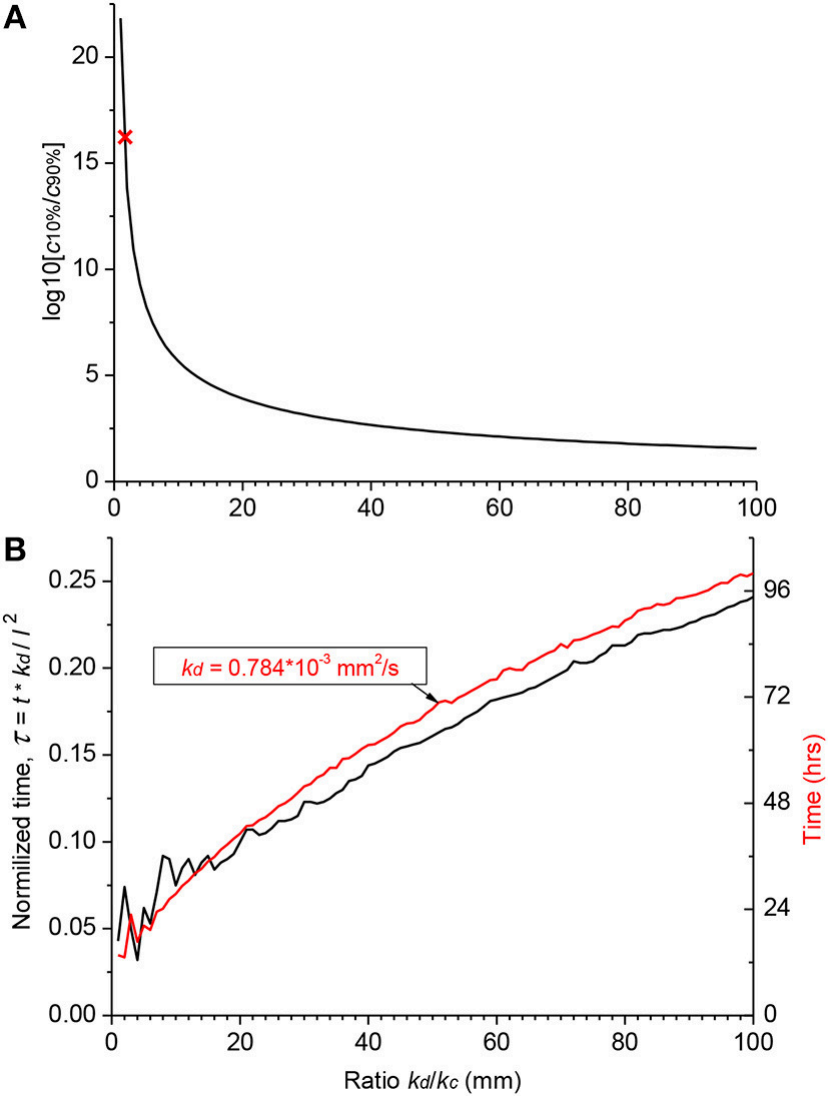
Theoretical distribution of an arbitrary substance in the human cochlea. **(A)** Dependence of the ratio of basal (*c*10%) and apical (*c*90%) steady-state concentrations on the ratio of diffusion (*k*_*d*_) and clearing (*k*_*c*_) coefficients. Red cross indicates the point for salicylate (*k*_*d*_/*k*_*c*_ = 1.7). **(B)** Normalized time (black curve, left ordinate) required to reach steady-state concentration at the cochlear apex for substances with different ratio of the diffusion (*k*_*d*_) and clearing (*k*_*c*_) coefficients. Red curve shows a specific example of the absolute time (right ordinate) for a substance with the diffusion coefficient (*k*_*d*_) similar to that of dexamethasone. The steady-state was defined as the normalized difference between consecutive numerical values of concentration <10^−4^. Jitter in the curves for small *k*_*d*_/*k*_*c*_ ratios is due to very low apical concentrations. The following geometrical parameters for human cochlea were used for calculations using the non-dimensional form of the diffusion equation (A14) *a* = 0.7981, *b* = 0.3990, and *l* = 28.46 mm (Thorne et al., 1999).

Hence, the validity of the diffusion/clearing equation has been confirmed using the experimental data on salicylate block of the cochlear amplifier, the equation can be used to make conclusions about the distribution of arbitrary substances along the human cochlea (Figure 4). Decrease in the relative contribution of clearing into the distribution of a substance along the ST, i.e.,. increase of *k*_*d*_/*k*_*c*_ ratio, leads to a dramatic reduction in the steady-state, base-to-apex gradient of the substance concentration (Figure 4A) calculated using the non-dimensional form of the diffusion equation (A14). This result is expected because a larger amount of the substance is available for diffusion into the cochlear apex in this case. For salicylate, however, the difference between the basal and apical concentrations is even larger in the human cochlea (red cross in Figure 4A) compared to guinea pigs and reaches 16 orders of magnitude because of the increased length of the human cochlea.

Figure 4A provides theoretical estimates of the minimal gradients which can be reached along the ST due to passive diffusion, when substances are in contact with the RW long enough to establish a concentration equilibrium distribution. Reduction in the base-to-apex gradient for substances with higher *k*_*d*_/*k*_*c*_ ratios, which are better retained in the ST, comes at the expense of the much longer substance exposure times required to reach steady-state concentration gradients (Figure 4B). For example, for a drug with the diffusion coefficient *k*_*d*_ similar to dexamethasone, for which the clearing coefficient is unknown, it takes days of retention at the RW when realistic *k*_*d*_/*k*_*c*_ ratios are assumed (red curve in Figure 4B). The problem is that, while it is theoretically possible to achieve smaller base-to apex concentration gradients for a drug with high *k*_*d*_/*k*_*c*_ ratios, in practice, if the drug is active, it will be cleared from the ST into the cochlear tissue, hence *k*_*c*_ cannot be arbitrarily small. In this case, the minimal theoretical difference in the base-to-apex concentrations of the drug is still a few orders of magnitude.

## Discussion

The existence of a base-to-apex drug concentration gradient, when drugs are applied to the RW, has been well-established. From this point of view, this study quantifies these gradients for the intact cochlea when the flow of perilymph in the ST is very small (Ohyama et al., 1988). This study does not investigate the problem of the RW permeability which is a separate challenge and requires specific considerations for particular drugs and formulations (Salt and Plontke, 2018). Instead, sodium salicylate which easily passes through the RW was used to ensure high concentrations at the cochlear base. Though, passive proton-mediated diffusion of salicylate across biomembranes is observed at micromolar concentrations (Takagi et al., 1998), the RW diffusional barrier could, presumably, be overcome by the drug at the much higher, submolar concentrations used in this study. While the RW membrane is highly permeable to salicylate and the CAP threshold elevation at high frequencies started within seconds after salicylate application, the model assumption that salicylate concentrations on both RW sides were the same might introduce some error in the calculated absolute concentrations. We would like to emphasize, however, that an error in calculation of the absolute concentrations (note that the absolute concentrations were calculated using Hallworth's (1997) empirical dependence between salicylate concentration and the OHC force reduction) does not lead to an error in calculation of the concentration gradient which is the basis for the conclusions in this study. This is true because the flux *J* is proportional to the concentration gradient [equation (A1)] and gradient curves calculated for different salicylate concentrations at the RW are scaled versions of each other with the same gradients. It is worth noting that, from discoveries we made in our preliminary experiments, salicylate concentrations higher than 100 mM used to study diffusion in this work caused elevation of CAP thresholds throughout the entire frequency range. This flooding of the whole cochlea with salicylate was due apparently to overloading of the cochlear clearing and other possible mechanisms involved. In this case, the dynamic equilibrium between diffusion and clearing and steady-state salicylate concentrations cannot be reached and our model cannot be applied. From an experimental standpoint, the use of higher concentrations of salicylate also made time-dependent estimates of diffusion impossible for high frequencies because the clearing mechanism in the basal turn became almost immediately saturated following salicylate application. As a result of the clearing overload and other unidentified processes, salicylate is accumulated throughout the cochlea affecting all the frequencies as it was observed in our experiments where we applied 1M salicylate to the RW. Of course, therapeutic use of concentrated drug formulations in order to overcome the issues raised by this study could be problematic due to likely side effects and/or restricted aqueous solubility and thus is not a practical solution.

While the steady-state distribution of concentrations, which is the basis for conclusions in this study, is fitted well by the simple diffusion model, the responses for the lower frequencies became gradually slower compared to the model predictions (Figure S1). This may happen because salicylate action on the cochlear amplifier is not limited by its block of the OHC motility (e.g., Russell and Schauz, 1995; Wu et al., 2010) as it is assumed in the model. A compensatory effect from a hypothetical mechanism maintaining cochlear homeostasis and OHC sensitivity and responsible for the “bounce” phenomenon after exposure to loud sounds (Kirk et al., 1997; Drexl et al., 2014) may also explain delayed threshold elevation at subtle salicylate concentrations in the low-frequency cochlear region. Finally, salicylate concentration at the cochlear base may be diluted by the cerebrospinal fluid coming through the cochlear aqueduct into the perilymph which becomes hyperosmotic due to relatively high salicylate concentration at the base. None of these mechanisms should, however, affect our conclusion about the magnitude of the steady-state concentration gradients along the ST.

For a cochlea of given geometry, the concentration gradient along the ST depends only on the relationship between diffusion and clearing and is drug specific. In terms of the current study, it is the value of *k*_*d*_/*k*_*c*_, which defines the ratio between the amount of drug entering through a unit surface of the ST normal to the direction of diffusion and leaving it through a unit area of the side walls within the same time period. Salicylate, which is readily cleared from the ST (*k*_*d*_/*k*_*c*_ = 1.6968), does not in practice diffuse into the cochlear apex and the resultant theoretical base-to-apex concentration gradient is extremely high (red cross in Figure 4A). Drugs which are better retained in the ST (i.e., have higher *k*_*d*_/*k*_*c*_ ratio) form smaller concentration gradients, but this is traded for the considerably longer time it takes for these drugs to reach steady-state concentrations in the cochlear apex (Figure 4B). Hence, this approach may not be practical when there is only a short time window for the treatment of a specific cochlear disorder. Also, using a drug form which is better retained in the ST will lead to larger concentration differences between the ST and surrounding tissue. This may be a problem for drugs with narrow therapeutic windows unless an inactive form of the drug is used for even distribution along the ST through diffusion and it is activated only when the drug is cleared into the surrounding tissue.

Because the retention of a drug at the RW does not lead to a leveling of its concentration along the cochlear spiral (see also Plontke et al., 2007b), different strategies for drug delivery to the cochlear apex should be employed. Stable drug loaded nanocarriers (Zou et al., 2014; Li et al., 2017; Kamalov et al., 2018) which can stay in the ST long enough without being cleared into the surrounding tissue may be a feasible option. When the concentration of nanocarriers along the ST reaches a constant level, the encapsulated drug could be released from the carriers through thermal or light activation (Karimi et al., 2016, 2017; Yuan et al., 2017) to obtain sufficient drug concentrations along the entire cochlear spiral. A potential problem with this approach is the substantial increase in time required to reach the equilibrium base-to-apex gradient of nanocarrier concentrations, due to the substantially smaller diffusion coefficients of even the smallest liposomes and micelles, compared to lone drug molecules (Figure 4B) (del Amo et al., 2017).

Drug loaded nanoparticles, however, could be used to take advantage of anatomical and cellular features of the cochlea which enable drug uptake through routes and pathways other than the ST route (Glueckert et al., 2018). Disulfiram loaded nanoparticles, for example, were observed in the apical part of the spiral ganglion just 1 day after their application to the RW and elevation of auditory brainstem response thresholds, due to disulfiram induced apoptosis of the ganglion neurons, was detected for frequencies corresponding to the cochlear apex within 2 days after application (Buckiová et al., 2012). Nanoparticles can also be effectively driven and distributed along the entire cochlea. Assisted diffusion of magnetically driven, prednisolone-loaded magnetic nanoparticle along the cochlea resulted in a significant increase in the protective effect of the drug against cisplatin-induced ototoxicity compared to intratympanic injections of prednisolone (Ramaswamy et al., 2017).

This study investigates passive drug diffusion along the intact cochlea when the drug is applied to the RW and highlights intrinsic problems with this method of local drug administration into the inner ear. Retaining drugs at the RW for an arbitrarily long time does not decrease its base-to-apex concentration gradient, which, at steady state, depends solely upon the relationship between drug diffusion along and clearing from the ST. Usage of drug-loaded nanocarriers which utilize the anatomical and cellular properties of the cochlea, and which can be actively distributed along the entire length of the cochlea seems to be a more promising approach.

## Ethics Statement

All procedures involving animals were performed in accordance with UK Home Office regulations with approval from the University of Brighton Animal Welfare and Ethical Review Body.

## Author Contributions

AL, IR, and TA conceived and designed the study. GB, SF, and AL performed the experiments and analyzed experimental results. IS, JK, and AL developed the model. IS performed numerical simulations and fitting to the experimental data. All authors contributed to analysis and discussion of the results. IS and AL wrote the manuscript with contribution from all authors.

### Conflict of Interest Statement

The authors declare that the research was conducted in the absence of any commercial or financial relationships that could be construed as a potential conflict of interest.

## Appendix

Only diffusion along the long axis *x* of the tube of decreasing diameter is considered (Figure 1A). The concentration *c* within each cross-section for a fixed instance *t* is assumed to be constant, i.e., *c* = *c*(*x, t*) is independent to the *y* axis. If the area of the cross-section is *S*(*x*), then the flux *J* along the *x* axis is given by:

(A1) $$\begin{array}{l} J\left(x,t\right)=-S\left(x\right)\cdot k_{d}\cdot \frac{dc\left(x,t\right)}{dx}, \end{array}$$

where *k*_*d*_ is the diffusion coefficient.

At the same time the clearing of salicylate from the tube of length Δ*x*, its perimeter *P*(*x*) and with an area of surface *P*(*x*)Δ*x*, can be described as:

(A2) $$\begin{array}{l} Cl\left(x,t\right)=c\left(x,t\right)\cdot k_{c}\cdot P\left(x\right)\cdot \Delta x, \end{array}$$

where *k*_*c*_ is the clearing coefficient.

The balance of fluxes and clearing in the volume between *x*_0_ and *x*_1_ can be described as:

(A3) $$\begin{array}{l} S\left(x\right)\cdot \Delta x\cdot \frac{dc\left(x,t\right)}{dt}=S\left(x_{1}\right)\cdot k_{d}\cdot \frac{dc\left(x_{1},t\right)}{dx}-S\left(x_{0}\right)\cdot k_{d}\cdot \frac{dc\left(x_{0},t\right)}{dx}\\ -c\left(x,t\right)\cdot k_{c}\cdot P\left(x\right)\cdot \Delta x, \end{array}$$

where Δ*x* = *x*_1_ − *x*_0_ is positive and *x* is in [*x*_0_, *x*_1_].

Divide both sides of Equation (A3) by Δ*x* and rearrange it as follows:

(A4) $$\begin{array}{l} S\left(x\right)\cdot \frac{dc\left(x,t\right)}{dt}=\frac{S\left(x_{1}\right)\cdot k_{d}\cdot dc\left(x_{1},t\right)/dx-S\left(x_{0}\right)\cdot k_{d}\cdot dc\left(x_{0},t\right)/dx}{\Delta x}\\ -c\left(x,t\right)\cdot k_{c}\cdot P\left(x\right). \end{array}$$

Take the limit of Δ*x* converging to zero and divide by *S*(*x*), the following is obtained:

(A5) $$\begin{array}{l} \frac{dc\left(x,t\right)}{dt}=\frac{1}{S\left(x\right)}\cdot \frac{d}{dx}\left(S\left(x\right)\cdot k_{d}\cdot \frac{dc\left(x,t\right)}{dx}\right)-c\left(x,t\right)\cdot L\left(x\right), \end{array}$$

where $L\left(x\right)=k_{c}\cdot \frac{P\left(x\right)}{S\left(x\right)}$ is an integral coefficient of clearing.

The perimeter is

(A6) $$\begin{array}{l} P\left(x\right)=2\pi \cdot \left(mx+a\right) \end{array}$$

and the area is

(A7) $$\begin{array}{l} S\left(x\right)=\pi \cdot {\left(mx+a\right)}^{2}, \end{array}$$

where *m* = (*b* − *a*)/*l*.

The integral coefficient can be written as:

(A8) $$\begin{array}{l} L\left(x\right)=k_{c}\cdot \frac{P\left(x\right)}{S\left(x\right)}=\frac{2k_{c}}{r\left(x\right)}. \end{array}$$

Thus, the diffusion can be described by the following partial differential equation

(A9) $$\begin{array}{l} \frac{dc\left(x,t\right)}{dt}=\frac{1}{S\left(x\right)}\cdot \frac{d}{dx}\left(S\left(x\right)\cdot k_{d}\cdot \frac{dc\left(x,t\right)}{dx}\right)-c\left(x,t\right)\cdot \frac{2k_{c}}{r\left(x\right)}, \end{array}$$

with the boundary conditions

(A10) $$\begin{array}{lll} c\left(0,t\right) & = & c_{rw}, \end{array}$$

(A11) $$\begin{array}{lll} k_{d}\frac{dc\left(l,t\right)}{dx} & = & 0 \end{array}$$

and initial conditions

(A12) $$\begin{array}{lll} c\left(0,0\right) & = & c_{rw};x=0, \end{array}$$

(A13) $$\begin{array}{lll} c\left(x,0\right) & = & 0;x>0. \end{array}$$

Equation (A9) can be rewritten in the following non-dimensional form

(A14) $$\begin{array}{l} \frac{du\left(\chi ,\tau \right)}{d\tau }=\frac{1}{{\left(\left(b-a\right)\chi +a\right)}^{2}}\cdot \frac{d}{d\chi }\left({\left(\left(b-a\right)\chi +a\right)}^{2}\cdot \frac{du\left(\chi ,\tau \right)}{d\chi }\right)\\ -u\left(\chi ,\tau \right)\cdot \frac{2l^{2}/ratio}{\left(b-a\right)\chi +a}, \end{array}$$

with the boundary conditions

(A15) $$\begin{array}{lll} u\left(0,\tau \right) & = & 1, \end{array}$$

(A16) $$\begin{array}{lll} ratio\cdot \frac{du\left(1,\tau \right)}{d\chi } & = & 0 \end{array}$$

and the initial conditions

(A17) $$\begin{array}{l} c\left(0,0\right)=1;\chi =0, \end{array}$$

(A18) $$\begin{array}{l} c\left(\chi ,0\right)=0;\chi >0, \end{array}$$

where *u* = *c*/*c*_*rw*_, $\tau =t\cdot k_{d}/l^{2}$ and χ = *x*/*l*.

## References

[B1] Borkholder D. A. Zhu X. Frisina R. D. (2014). Round window membrane intracochlear drug delivery enhanced by induced advection. J. Control. Release 174, 171–176. 10.1016/j.jconrel.2013.11.021 24291333 PMC3925065

[B2] Borkholder D. A. Zhu X. Hyatt B. T. Archilla A. S. Livingston I. I. I. W. J. III Frisina R. D. (2010). Murine intracochlear drug delivery: reducing concentration gradients within the cochlea. Hear. Res. 268, 2–11. 10.1016/j.heares.2010.04.014 20451593 PMC2933796

[B3] Bowe S. N. Jacob A. (2010). Round window perfusion dynamics: implications for intracochlear therapy. Curr. Opin. Otolaryngol. Head Neck Surg. 18, 377–385. 10.1097/MOO.0b013e32833d30f0 20808222

[B4] Buckiová D. Ranjan S. Newman T. A. Johnston A. H. Sood R. Kinnunen P. K.. (2012). Minimally invasive drug delivery to the cochlea through application of nanoparticles to the round window membrane. Nanomedicine 7, pp., 1339–1354. 10.2217/nnm.12.5 22475648

[B5] Chen Z. Kujawa S. G. McKenna M. J. Fiering J. O. Mescher M. J. Borenstein J. T.. (2005). Inner ear drug delivery via a reciprocating perfusion system in the guinea pig. J. Control. Release 110, pp., 1–19. 10.1016/j.jconrel.2005.09.003 16274830 PMC2030590

[B6] Creber N. J. Eastwood H. T. Hampson A. J. Tan J. O'Leary S. J. (2018). A comparison of cochlear distribution and glucocorticoid receptor activation in local and systemic dexamethasone drug delivery regimes. Hear. Res. 368, 75–85. 10.1016/j.heares.2018.03.018 29622283

[B7] del Amo E. M. Rimpelä A. K. Heikkinen E. Kari O. K. Ramsay E. Lajunen T.. (2017). Pharmacokinetic aspects of retinal drug delivery. Prog. Retin. Eye Res. 57, 134–185. 10.1016/j.preteyeres.2016.12.001 28028001

[B8] Devare J. Gubbels S. Raphael Y. (2018). Outlook and future of inner ear therapy. Hear. Res. 368, 127–135. 10.1016/j.heares.2018.05.009 29804723 PMC6165678

[B9] Drexl M. Überfuhr M. Weddell T. D. Lukashkin A. N. Wiegrebe L. Krause E.. (2014). Multiple indices of the ‘bounce’ phenomenon obtained from the same human ears. J. Assoc. Res. Otolaryngol. 15, 57–72. 10.1007/s10162-013-0424-x 24253659 PMC3901855

[B10] El Kechai N. Agnely F. Mamelle E. Nguyen Y. Ferrary E. Bochot A. (2015). Recent advances in local drug delivery to the inner ear. Int. J. Pharm. 494, 83–101. 10.1016/j.ijpharm.2015.08.015 26260230

[B11] Glueckert R. Chacko L. J. Rask-Andersen H. Wei L. Handschuh S. Schrott-Fischer A. (2018). Anatomical basis of drug delivery to the inner ear. Hear. Res. 368, 10–27. 10.1016/j.heares.2018.06.017 30442227

[B12] Greenwood D. D. (1990). A cochlear frequency-position function for several species−29 years later. J. Acoust. Soc. Am. 87, 2592–2605. 10.1121/1.399052 2373794

[B13] Grewal A. S. Nedzelski J. M. Chen J. M. Lin V. Y. (2013). Dexamethasone uptake in the murine organ of Corti with transtympanic versus systemic administration. J. Otolaryngol.-Head N. 42:19. 10.1186/1916-0216-42-19 23663237 PMC3651220

[B14] Haghpanahi M. Gladstone M. B. Zhu X. Frisina R. D. Borkholder D. A. (2013). Noninvasive technique for monitoring drug transport through the murine cochlea using micro-computed tomography. Ann. Biomed. Eng. 41, 2130–2142. 10.1007/s10439-013-0816-4 23636576 PMC6434528

[B15] Hallworth R. (1997). Modulation of outer hair cell compliance and force by agents that affect hearing. Hear. Res. 114, 204–212. 10.1016/S0378-5955(97)00167-6 9447933

[B16] Hao J. Li S. K. (2019). Inner ear drug delivery: recent advances, challenges, and perspective. Eur. J. Pharm. Sci. 126, 82–92. 10.1016/j.ejps.2018.05.020 29792920

[B17] Hargunani C. A. Kempton J. B. DeGagne J. M. Trune D. R. (2006). Intratympanic injection of dexamethasone: time course of inner ear distribution and conversion to its active form. Otol. Neurotol. 27, 564–569. 10.1097/01.mao.0000194814.07674.4f 16691147

[B18] Imamura S. I. Adams J. C. (2003). Distribution of gentamicin in the guinea pig inner ear after local or systemic application. J. Assoc. Res. Otolaryngol. 4, 176–195. 10.1007/s10162-002-2036-8 12943372 PMC3202710

[B19] Jones G. P. Lukashkina V. A. Russell I. J. Elliott S. J. Lukashkin A. N. (2013). Frequency-dependent properties of the tectorial membrane facilitate energy transmission and amplification in the cochlea. Biophys. J. 104, 1357–1366. 10.1016/j.bpj.2013.02.002 23528095 PMC3602785

[B20] Kamalov M. I. Dǎng T. Petrova N. V. Laikov A. V. Luong D. Akhmadishina R. A.. (2018). Self-assembled nanoformulation of methylprednisolone succinate with carboxylated block copolymer for local glucocorticoid therapy. Colloids Surf. B Biointerfaces 164, pp. 78–88. 10.1016/j.colsurfb.2018.01.014 29413623

[B21] Karimi M. Sahandi Zangabad P. Baghaee-Ravari S. Ghazadeh M. Mirshekari H. Hamblin M. R. (2017). Smart nanostructures for cargo delivery: uncaging and activating by light. J. Am. Chem. Soc. 139, 4584–4610. 10.1021/jacs.6b08313 28192672 PMC5475407

[B22] Karimi M. Sahandi Zangabad P. Ghasemi A. Amiri M. Bahrami M. Malekzad H.. (2016). Temperature-responsive smart nanocarriers for delivery of therapeutic agents: applications and recent advances. ACS Appl. Mater. Interfaces 8, 21107–21133. 10.1021/acsami.6b00371 27349465 PMC5003094

[B23] Kirk D. L. Moleirinho A. Patuzzi R. B. (1997). Microphonic and DPOAE measurements suggest a micromechanical mechanism for the ‘bounce’ phenomenon following low-frequency tones. Hear. Res. 112, 69–86. 10.1016/S0378-5955(97)00104-4 9367230

[B24] Li L. Chao T. Brant J. O'Malley B. Jr Tsourkas A. Li D. (2017). Advances in nano-based inner ear delivery systems for the treatment of sensorineural hearing loss. Adv. Drug Deliv. Rev. 108, 2–12. 10.1016/j.addr.2016.01.004 26796230 PMC4940320

[B25] Lide D. R. (2002). CRC Handbook of Chemistry and Physics, 83rd Edn. Boca Raton, FL: CRC Press.

[B26] Lukashkin A. N. Lukashkina V. A. Russell I. J. (2002). One source for distortion product otoacoustic emissions generated by low- and high-level primaries. J. Acoust. Soc. Am. 111, 2740–2748. 10.1121/1.1479151 12083209

[B27] Lukashkin A. N. Russell I. J. (1999). Analysis of the f2– f1 and 2f1– f2 distortion components generated by the hair cell mechanoelectrical transducer: Dependence on the amplitudes of the primaries and feedback gain. J. Acoust. Soc. Am. 106, 2661–2668. 10.1121/1.428096

[B28] Meaud J. Grosh K. (2014). Effect of the attachment of the tectorial membrane on cochlear micromechanics and two-tone suppression. Biophys. J. 106, 1398–1405. 10.1016/j.bpj.2014.01.034 24655515 PMC3984990

[B29] Mountain D. C. Hubbard A. E. McMullen T. A. (1983). Electromechanical processes in the cochlea, in Mechanics of Hearing, eds. de Boer E. Viergever M. A. (Dordrecht: Springer), 119–126.

[B30] Mynatt R. Hale S. A. Gill R. M. Plontke S. K. Salt A. N. (2006). Demonstration of a longitudinal concentration gradient along scala tympani by sequential sampling of perilymph from the cochlear apex. J. Assoc. Res. Otolaryngol. 7, 182–193. 10.1007/s10162-006-0034-y 16718612 PMC1945159

[B31] Ni G. Elliott S. J. Baumgart J. (2016). Finite-element model of the active organ of Corti. J. R. Soc. Interface 13:20150913. 10.1098/rsif.2015.0913 26888950 PMC4780563

[B32] Nuttall A. L. Ricci A. J. Burwood G. Harte J. M. Stenfelt S. Cayé-Thomasen P.. (2018). A mechanoelectrical mechanism for detection of sound envelopes in the hearing organ. Nat. Commun. 9:4175. 10.1038/s41467-018-06725-w 30302006 PMC6177430

[B33] Ohyama K. Salt A. N. Thalmann R. (1988). Volume flow rate of perilymph in the guinea-pig cochlea. Hear. Res. 35, 119–129. 10.1016/0378-5955(88)90111-6 3198505

[B34] Plontke S. K. Biegner T. Kammerer B. Delabar U. Salt A. N. (2008). Dexamethasone concentration gradients along scala tympani after application to the round window membrane. Otol. Neurotol. 29, 401–406. 10.1097/MAO.0b013e318161aaae 18277312 PMC2587453

[B35] Plontke S. K. Mynatt R. Gill R. M. Borgmann S. Salt A. N. (2007a). Concentration gradient along the scala tympani after local application of gentamicin to the round window membrane. Laryngoscope, 117, 1191–1198. 10.1097/MLG.0b013e318058a06b 17603318 PMC2728588

[B36] Plontke S. K. Siedow N. Wegener R. Zenner H. P. Salt A. N. (2007b). Cochlear pharmacokinetics with local inner ear drug delivery using a three-dimensional finite-element computer model. Audiol. Neurotol. 12, 37–48. 10.1159/000097246 17119332 PMC1779502

[B37] Ramaswamy B. Roy S. Apolo A. B. Shapiro B. Depireux D. A. (2017). Magnetic nanoparticle mediated steroid delivery mitigates cisplatin induced hearing loss. Front. Cell. Neurosci. 11:268. 10.3389/fncel.2017.00268 28955202 PMC5601400

[B38] Rivera T. Sanz L. Camarero G. Varela-Nieto I. (2012). Drug delivery to the inner ear: strategies and their therapeutic implications for sensorineural hearing loss. Curr. Drug Deliv. 9, 231–242. 10.2174/156720112800389098 22283653

[B39] Ruggero M. A. Narayan S. S. Temchin A. N. Recio A. (2000). Mechanical bases of frequency tuning and neural excitation at the base of the cochlea: comparison of basilar-membrane vibrations and auditory-nerve-fiber responses in chinchilla. Proc. Natl. Acad. Sci. U.S.A. 97, 11744–11750. 10.1073/pnas.97.22.11744 11050204 PMC34344

[B40] Russell I. J. Schauz C. (1995). Salicylate ototoxicity: effects on stiffness and electromotility of outer hair cells isolated from the guinea pig cochlea. Auditory Neurosci. 1, 309–319.

[B41] Saijo S. Kimura R. S. (1984). Distribution of HRP in the inner ear after injection into the middle ear cavity. Acta Otolaryngol. 97, 593–610. 10.3109/00016488409132937 6464711

[B42] Salt A. N. Hirose K. (2018). Communication pathways to and from the inner ear and their contributions to drug delivery. Hear. Res. 362, 25–37. 10.1016/j.heares.2017.12.010 29277248 PMC5911243

[B43] Salt A. N. Kellner C. Hale S. (2003). Contamination of perilymph sampled from the basal cochlear turn with cerebrospinal fluid. Hear. Res. 182, 24–33. 10.1016/S0378-5955(03)00137-0 12948598

[B44] Salt A. N. Ma Y. (2001). Quantification of solute entry into cochlear perilymph through the round window membrane. Hear. Res. 154, 88–97. 10.1016/S0378-5955(01)00223-4 11423219

[B45] Salt A. N. Plontke S. K. (2009). Principles of local drug delivery to the inner ear. Audiol. Neurotol. 14, 350–360. 10.1159/000241892 19923805 PMC2820328

[B46] Salt A. N. Plontke S. K. (2018). Pharmacokinetic principles in the inner ear: influence of drug properties on intratympanic applications. Hear. Res. 368, 28–40. 10.1016/j.heares.2018.03.002 29551306 PMC6133771

[B47] Schuknecht H. F. (1956). Ablation therapy for the relief of Meniere's disease. Laryngoscope 66, 859–859. 10.1288/00005537-195607000-00005 13358249

[B48] Takagi M. Taki Y. Sakane T. Nadai T. Sezaki H. Oku N. (1998). A new interpretation of salicylic acid transport across the lipid bilayer: implications of pH-dependent but not carrier-mediated absorption from the gastrointestinal tract. J. Pharmacol. Exp. Ther. 285, 1175–1180. 9618420

[B49] Temchin A. N. Rich N. C. Ruggero M. A. (2008). Threshold tuning curves of chinchilla auditory-nerve fibers. I. Dependence on characteristic frequency and relation to the magnitudes of cochlear vibrations. J. Neurophysiol. 100, 2889–2898. 10.1152/jn.90637.2008 18701751 PMC2585409

[B50] Thorne M. Salt A. N. DeMott J. E. Henson M. M. Henson O. W. Jr. Gewalt S. L. (1999). Cochlear fluid space dimensions for six species derived from reconstructions of three-dimensional magnetic resonance images. Laryngoscope 109, 1661–1668. 10.1097/00005537-199910000-00021 10522939

[B51] Wu T. Lv P. Kim H. J. Yamoah E. N. Nuttall A. L. (2010). Effect of salicylate on KCNQ4 of the guinea pig outer hair cell. J. Neurophysiol. 103, 1969–1977. 10.1152/jn.01057.2009 20147414 PMC2853271

[B52] Yates G. K. (1990). Basilar membrane nonlinearity and its influence on auditory nerve rate-intensity functions. Hear. Res. 50, 145–162. 10.1016/0378-5955(90)90041-M 2076968

[B53] Yuan A. Huan W. Liu X. Zhang Z. Zhang Y. Wu J.. (2017). NIR light-activated drug release for synergetic chemo-photothermal therapy. Mol. Pharm. 14, 242–251. 10.1021/acs.molpharmaceut.6b00820 27983855

[B54] Zou J. Sood R. Zhang Y. Kinnunen P. K. Pyykkö I. (2014). Pathway and morphological transformation of liposome nanocarriers after release from a novel sustained inner-ear delivery system. Nanomedicine 9, 2143–2155. 10.2217/nnm.13.181 24471501

