# Supplementary material for: Drug Diffusion Along an Intact Mammalian Cochlea
Source: Front Cell Neurosci. 2019 Apr 26;13:161. doi: 10.3389/fncel.2019.00161 (PMC6497751; doi:10.3389/fncel.2019.00161)
Supplement: Supplementary file 1 [file Data_Sheet_1.PDF]

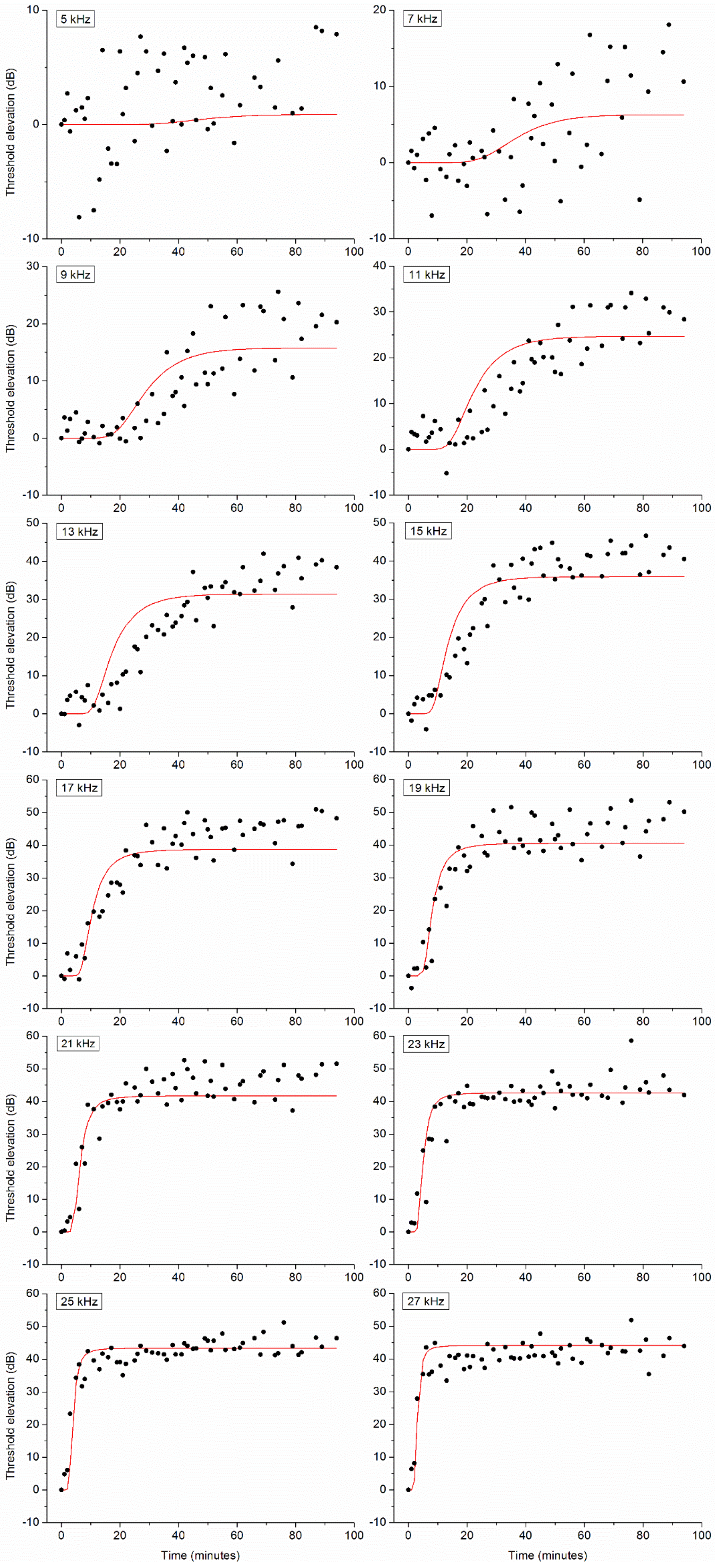
5 kHz
7 kHz
9 kHz
11 kHz
13 kHz
15 kHz
17 kHz
19 kHz
21 kHz
23 kHz
25 kHz
27 kHz
Threshold elevation (dB)
Time (minutes)

FIGURE S1. Pooled data for 5 animals (black circles) showing the CAP threshold elevation after application of 100 mM solution of salicylate to the RW at time = 0. Red curves were fitted to the entire set experimental data using general optimization procedure.
